# Atrial fibrillation, quality of life and distress: a cluster analysis of cognitive and behavioural responses

**DOI:** 10.1007/s11136-021-03006-w

**Published:** 2021-10-07

**Authors:** Elaina C. Taylor, Mark O’Neill, Lyndsay D. Hughes, Rona Moss-Morris

**Affiliations:** 1 Health Psychology Section, Institute of Psychiatry, Psychology and Neuroscience, King’s College London, 5th Floor Bermondsey Wing, Guy’s Hospital, London Bridge, London, SE1 9RT UK; 2 Divisions of Imaging Sciences & Biomedical Engineering & Cardiovascular Medicine, King’s College London, 4th Floor North Wing, St Thomas’ Hospital, London, UK; 3 University of Derby, Kedleston Road, Derby, DE22 1GB UK

**Keywords:** Atrial fibrillation, Quality of life, Anxiety, Depression, Illness perceptions, Cognitive and behavioural responses to symptoms, Cluster analysis

## Abstract

**Purpose:**

Few studies have examined specific cognitive and behavioural responses to symptoms, which may impact health-related outcomes, in conjunction with illness representations, as outlined by the Common-Sense-Model. Patients with atrial fibrillation (AF) report poor quality-of-life (QoL) and high distress. This cross-sectional study investigated patterns/clusters of cognitive and behavioural responses to illness, and illness perceptions, and relationships with QoL, depression and anxiety.

**Methods:**

AF patients (*N* = 198) recruited at cardiology clinics completed the AF-Revised Illness Perception Questionnaire, Atrial-Fibrillation-Effect-on-Quality-of-Life Questionnaire, Patient Health Questionnaire-8 and Generalized Anxiety Disorder Questionnaire. Cluster analysis used Ward’s and *K*-means methods. Hierarchical regressions examined relationships between clusters with QoL, depression and anxiety.

**Results:**

Two clusters of cognitive and behavioural responses to symptoms were outlined; (1) ‘high avoidance’; (2) ‘low symptom-focussing’. Patients in Cluster 1 had lower QoL (*M* = 40.36, SD = 18.40), greater symptoms of depression (*M* = 7.20, SD = 5.71) and greater symptoms of anxiety (*M* = 5.70, SD = 5.90) compared to patients in Cluster 2 who had higher QoL (*M* = 59.03, SD = 20.12), fewer symptoms of depression (*M* = 3.53, SD = 3.56) and fewer symptoms of anxiety (*M* = 2.56, SD = 3.56).

Two illness representation clusters were outlined; (1) ‘high coherence and treatment control’, (2) ‘negative illness and emotional representations’. Patients in Cluster 2 had significantly lower QoL (*M* = 46.57, SD = 19.94), greater symptoms of depression (*M* = 6.12, SD = 5.31) and greater symptoms of anxiety (*M* = 4.70, SD = 5.27), compared with patients in Cluster 1 who had higher QoL (*M* = 61.52, SD = 21.38), fewer symptoms of depression (*M* = 2.85, SD = 2.97) and fewer symptoms of anxiety (*M* = 2.16, SD = 3.63).

Overall, clusters of cognitive and behavioural responses to symptoms, and illness perceptions significantly explained between 14 and 29% of the variance in QoL, depression and anxiety.

**Conclusion:**

Patterns of cognitive and behavioural responses to symptoms, and illness perceptions are important correlates of health-related outcomes in AF patients.

## Plain English summary

Individuals with atrial fibrillation (AF) experience poor quality of life (QoL). Previous research in AF patients suggests the importance of individual illness-specific cognitions and behaviours in impacting patient-reported outcomes. However, no previous research has identified specific patterns of illness-related cognition or behaviours in AF patients, and how these might be related to QoL and distress. Using cluster analysis, this study examined whether specific patterns of illness-related cognitions and behaviours existed, and whether these patterns were related to differences in QoL, anxiety and depression.

This study found two illness-related cognitive patterns and two behavioural patterns of responding to AF. Patients who had a greater perceived understanding of their illness and treatment had greater QoL and lower distress than people who had more negative perceptions of their illness (such as that AF has greater consequences). Individuals who were more likely to avoid activities or engage in excessive rest due to their symptoms had greater distress and poorer QoL, than individuals who focussed less on their symptoms and reported less behavioural avoidance.

Findings from this study indicate the importance of identifying patterns of illness-related cognitions and behaviours which may help identify those at greater risk of poor health-related outcomes in a clinical setting.

## Introduction

Atrial fibrillation (AF) is a common heart-rhythm disorder, characterised by symptoms including heart palpitations, shortness of breath and fatigue. AF patients experience high levels of distress (anxiety and depression) and poor quality of life (QoL) [1, 2]. Procedural treatments may successfully restore sinus rhythm, but they do not always show a concomitant improvement in distress or QoL [3–5].

Previous research suggests that the way AF patients view their illness (illness perceptions) may provide insight into poor QoL. For example, beliefs that AF had greater consequences on everyday life was associated with poorer QoL [6]. In addition, a qualitative study outlined the importance of patients’ behavioural responses to symptoms, in impacting health-related outcomes such as distress. Patients who reported beliefs that the symptoms of AF were unpredictable, also reported behavioural responses such as avoidance, excessive resting behaviours, or all-or-nothing behaviours (over-activity and underactivity), a perceived lack of control of self-management of symptoms, and heightened distress [6].

The Common-Sense Model (CSM) [7, 8] is a theoretical framework, used to understand how patients’ cognitive and behavioural responses to illness may inform health-related outcomes. The CSM suggests a dynamic bi-directional influence of illness perceptions on coping behaviours/behavioural responses to symptoms. Appraisal of the efficacy of behavioural responses to symptoms may inform outcomes such as distress and QoL. The CSM has been widely supported in cardiovascular illnesses including myocardial infarction and heart disease, and to a lesser extent in AF. However much of this research specifically relates to illness perceptions and general coping, and in comparison, very little research has been conducted into specific behavioural responses and potential associations with outcome [9–11].

Furthermore, in relation to methodology, illness perceptions are often conceptualised as individual items. For instance, these include illness identity (attributed symptoms), timeline (chronic/acute), cyclic timeline (recurrence of illness/symptoms), consequences (impact on the illness on everyday life), personal control (self-management of symptoms), treatment control (management of symptoms through pharmacological/surgical means), illness coherence (perceived understanding of the illness including symptoms, causes and management), and emotional representations of illness [8, 12]. However, the original CSM indicated that illness perceptions should be viewed as clusters, or patterns of beliefs, forming an illness schema or illness representation [8]. In line with Leventhal’s concept, there has been some effort to identify patterns of illness beliefs in other long-term conditions (LTCs), to develop more theoretically meaningful illness representation profiles [13, 14]. For example, McCorry et al. identified two illness perception clusters predicting distress in breast cancer patients [15]. Patients in Cluster 1 had stronger beliefs about chronic and cyclic timeline, more negative consequences, lower illness coherence and personal and treatment control than patients in Cluster 2. Cluster membership predicted 25% of the variance in anxiety symptoms at diagnosis and 10% of the variance after 6 months. Similarly, cluster membership predicted 21% of the variance in depressive symptoms at diagnosis and 11% after 6 months. These studies indicate that illness schemas may help to profile patients most at risk of negative outcomes such as distress and poor QoL [13, 16].

Similar to illness perceptions, behavioural responses to illness are commonly examined as individual items, rather than clusters. In line with the CSM, patients may have patterns of coping with, and managing, illness. Additionally, the majority of previous research examining patients’ responses to illness, focusses on more generic models of coping, i.e. the stress and coping paradigm proposed by Lazarus & Folkman, which outline broad coping responses which can be applied to general stress-related responses [17, 18]. In contrast, examining more specific illness-related responses [19] may more accurately capture idiosyncratic responses to AF.

To our knowledge, no previous research has examined illness representation profiles, or cognitive and behavioural responses to symptoms profiles in AF patients. Identifying AF-specific illness profiles associated with poor QoL, anxiety and depression, may allow at-risk patients to be targeted for intervention. This study aims to (1) examine possible illness representation clusters and specific behavioural responses clusters in AF patients and (2) examine the association between illness representation cluster and cognitive and behavioural responses to symptoms (CBRS) cluster with AF-specific QoL, anxiety and depression.

## Methods

### Participants and procedure

Participants were recruited from cardiology out-patient clinics and online from the Atrial Fibrillation Association (AFA) website. Patients diagnosed with persistent AF, who were fluent in English, and had no severe co-morbidities (classified as severe heart failure, active cancer, dementia or hospitalisation for diabetes in the past year) were included. Participants were given questionnaires in clinic or online to complete and returned in clinic/online. Of 246 eligible patients approached in clinic, 174 consented to participate (71% response rate) and a further 24 were recruited online; (*N* = 198). This cross-sectional study was part of a larger longitudinal study. Ethical approval was granted from the National Health Service Research Ethics Committee (London Bloomsbury REC: 14/LO/2148).

### Measures

Clinical and demographic variables including gender, age, treatment procedures due to be undertaken, and whether previous treatment procedures (catheter ablation or cardioversion) were obtained from patients’ questionnaires and medical notes. Patients who are more symptomatic seek procedural treatment [20]. We therefore included treatment-related variables to examine any potential impact on QoL and distress outcomes.

#### The Atrial Fibrillation Revised Illness Perception Questionnaire (AF-IPQ-R)

The Atrial Fibrillation Revised Illness Perception Questionnaire (AF-IPQ-R); [6] is a modified version of the Revised Illness Perception Questionnaire [12], specific to AF patients. The AF-IPQ-R has good construct validity and test–retest reliability [6]. The AF-IPQ-R components remain the same as the IPQ-R (identity, timeline (chronic/acute), cyclic timeline, treatment and personal control, consequences, coherence) except the causes scale was modified to a triggers scale consisting of emotional, health behaviours and over-exertion triggers. The treatment control component was also divided into subscales, relating to procedural- and pharmacological treatments. Satisfactory reliability was evidenced in the current sample *α* = .62.

#### The Cognitive and Behavioural Responses to Symptoms Questionnaire (CBRQ)

The Cognitive and Behavioural Responses to Symptoms Questionnaire (CBRQ) [21] is a 40-item questionnaire with two scales measuring cognitive and behavioural responses to symptoms on a five-point Likert-type scale (0 = never to 4 = all the time). Based on qualitative AF research and other previous studies, the embarrassment avoidance scale was removed, and the behavioural component divided into three subscales [22]. The CBRQ therefore consisted of four cognitive subscales (fear avoidance, catastrophising, damaging beliefs, and symptom focus) and three behavioural subscales (resting, avoidance and all-or-nothing behaviours). The CBRQ has evidenced good internal reliability [21] and showed good reliability in the current sample *α* = .92.

#### The Atrial Fibrillation Effect on Quality of Life (AFEQT)

The Atrial Fibrillation Effect on Quality of Life (AFEQT) [23] measures AF-specific QoL consisting of four scales examining; symptoms, activities, treatment concern and treatment satisfaction, providing an overall health score. A seven-point Likert-type scale (from ‘not at all’ to ‘extremely limited’) is used in all components. The AFEQT evidences good test–retest reliability and construct validity [24] and strong reliability in the current sample *α* = .76.

#### The Patient Health Questionnaire (PHQ-8)

The Patient Health Questionnaire (PHQ-8) [25] measures symptoms of depression, using 8 items omitting the ninth item on suicide of the PHQ-9 [26]. Scoring uses a four-point Likert-type scale, with a sum score of ≥ 10 indicating clinically relevant depression. It is used widely in patients with cardiovascular disease with high validity and reliability [25] including in the current sample (*α* = .88).

#### The Generalized Anxiety Disorder Questionnaire (GAD-7)

The Generalized Anxiety Disorder Questionnaire (GAD-7) [27] is a 7-item measure of anxiety symptoms, using a four-point Likert-type scale. A sum score of ≥ 10 indicates clinically relevant anxiety. The GAD-7 has evidenced good validity [27] and showed strong reliability in the current sample *α* = .92).

### Statistical analysis

An A-Priori power analysis was conducted for sample size estimation. Based on previous studies with a large effect size [15, 28], an alpha of 0.05, power of 0.95 and 7 predictors, the proposed sample size was *N* = 61. Mean imputation was used to account for missing data, as less than 5% of the data were missing [29]. The significance level of *p* < 0.05 was used throughout the analysis.

Cluster analyses were conducted in SPSS (Version 25). A two-step cluster analysis was conducted. Pre-clustering was first conducted with Ward’s method [30] to determine the number of clusters. As an agglomerative method, it clusters similar elements minimising the variance within clusters at each stage of grouping. Ward’s method uses a data-driven approach to develop the optimum number of clusters, rather than arbitrary choice [31]. With the number of clusters confirmed by pre-clustering, *K*-means analysis was subsequently conducted. *K*-means analysis is less sensitive to outliers compared with other clustering methods, maximises inter-group differences, minimises intra-group differences and separates observations into uniform groups [32, 33]. Previous research has also indicated that the two-step clustering method is most reliable in detecting the number of sub-groups, classification of observations to groups, and for replicability [34–36]. Specifically previous research has indicated that the two-step method combining hierarchical and non-hierarchical procedures is most effective in identifying patterns of illness representations across individuals [13]. *Z* scores were used and squared Euclidean distance was used as the proximity measure. The final clusters were discussed between internal and external researchers and determined by interpretability and previous research.

Following the cluster analysis, preliminary univariate analysis was examined; Independent samples *t*-tests examined whether there were significant differences between illness representation cluster and CBRS cluster for (1) QoL, (2) depression and (3) anxiety.

Finally, three hierarchical regression analyses were conducted relating to each outcome (1) QoL, (2) depression and (3) anxiety. Demographic and clinical variables including age, gender, whether patients had experienced previous cardioversions or catheter ablations, and upcoming procedural treatments, were entered into block one, illness representation clusters were entered into block two and CBRS clusters were entered into block three. Previous research has established significant associations between illness representations and patient-reported outcomes, but has examined coping responses to a lesser extent [19]. Entering illness representations first, and on a separate step, helps determine any additional contribution of coping responses in explaining the variance.

## Results

### Cluster analysis

Mean age of the sample was 64 years (SD = 9.0). The majority were white British (97%) and male (77%). For both illness representation, and CBRS cluster analyses when using Ward’s method, the agglomeration schedule did not exhibit a clear cut ‘dog-leg’ representing the number of clusters in the sample, however indicated change around the low numbers, suggestive of a small number of clusters. Similarly, the horizontal distance in the dendrogram indicated the number of clusters to be less than five, with most change between two and three, therefore the number of clusters was set at two.

As determined by Ward’s method, *K*-means analysis was conducted with two clusters for both the illness representation and CBRS cluster analyses. Tables 1 and 2 outline mean values and ANOVAs for each cluster analysis. In relation to illness representations, Cluster 1 was labelled, ‘high coherence and treatment control’ and Cluster 2 was labelled ‘negative illness and emotional representations. Patients in ‘high coherence and treatment control’ group (*N* = 44) seemed to have more overall positive beliefs about their AF than patients in the ‘negative illness and emotional representations’ group. They were characterised by higher illness coherence beliefs, lower perceptions that AF was cyclic and lower scores on perceived triggers of AF. The triggers scale related to beliefs that AF symptoms could be triggered by emotional factors, such as stress, health behaviour/illness factors, such as other cardiovascular disease or smoking, or overexertion factors, including pushing themselves too hard. Patients in the ‘high coherence and treatment control’ group also held stronger beliefs about pharmacological treatment control (i.e. anticoagulants, but not procedural treatment control) in comparison to patients in the ‘negative illness and emotional representations’ group. Conversely, patients categorised into ‘negative illness and emotional representations’ (*N* = 100) attributed more symptoms to AF (illness identity), had more negative emotional representations about AF, stronger beliefs that AF was cyclic, had greater consequences, and higher levels of personal control, than patients in ‘high coherence and treatment control’. Patients in the ‘negative illness and emotional representations’ group scored highly on all trigger items, but particularly in relation to health-behaviour/illness triggers (including triggers such as alcohol, smoking, medication, other cardiovascular disease and diet).

**Table 1:** Illness representation clusters

	Total sample mean (SD)	High coherence and treatment control Mean (SD) *N* = 84	Negative illness and emotional representations Mean (SD) *N* = 144	*F*	Sig.
Illness identity	6.68 (3.10)	5.36 (2.89)	7.24(2.98)	16.48	< .001
Timeline (chronic/acute)	17.34 (4.15)	16.36 (4.27)	17.75 (4.04)	4.68	.126
Consequences	20.14 (4.24)	18.03 (4.85)	21.01 (3.65)	22.29	< .001
Personal control	14.24 (3.57)	12.69 (3.94)	14.88 (3.21)	16.63	< .001
Illness coherence	16.57 (4.53)	17.91 (5.66)	16.01 (3.86)	7.50	.002
Cyclic timeline	10.87 (3.53)	8.47 (3.06)	11.87 (3.22)	47.10	< .001
Emotional representations	20.09 (4.49)	16.91 (4.90)	21.40 (3.58)	51.42	< .001
Emotional triggers	13.86 (4.49)	9.71 (3.21)	15.59 (3.13)	142.61	< .001
Health behaviour triggers	19.14 (5.31)	14.10 (5.12)	21.22 (3.77)	117.11	< .001
Overexertion triggers	9.32 (2.65)	7.29 (2.66)	10.16 (2.14)	63.24	< .001
Treatment control (anticoagulant)	18.25 (2.58)	19.24 (2.58)	17.84 (2.47)	12.79	.007
Treatment control (procedures)	17.35 (2.45)	17.76 (2.92)	17.19 (2.22)	2.26	.092

*CBRS* Cognitive and behavioural responses to symptoms items

**Table 2:** Cognitive and behavioural responses to illness cluster

	Total sample mean (SD)	High avoidance cluster Mean (SD) *N* = 84	Low symptom-focussing and avoidance cluster Mean (SD) *N* = 114	*F*	Sig.
Fear avoidance	18.04 (4.36)	21.37 (3.09)	15.58 (3.47)	150.23	< 0.001
Catastrophising	8.13 (2.41)	8.93 (2.49)	7.54 (2.18)	17.38	< 0.001
Damaging	15.91 (3.33)	18.00 (2.97)	14.36 (2.67)	81.60	< 0.001
Symptom focus	17.82 (4.75)	20.54 (4.17)	15.82 (4.12)	62.67	< 0.001
All-or-nothing	10.37 (3.90)	12.28 (4.20)	8.96 (2.97)	42.41	< 0.001
Avoidance	13.22 (5.20)	17.62 (4.47)	9.98 (2.74)	220.26	< 0.001
Resting	9.65 (3.32)	12.04 (3.18)	7.89 (2.10)	122.37	< 0.001

In relation to CBRS clusters, Cluster 1 was labelled ‘high avoidance’. Patients in the ‘high avoidance’ cluster (*N* = 84) had more negative cognitive responses to symptoms and engaged in all of the unhelpful symptom behaviours. The cognitive pattern most distinct to the ‘high avoidance’ cluster was fear avoidance and the most dominant behaviours were avoidance and resting.

Cluster 2 was labelled ‘low symptom-focussing and avoidance’. Patients in Cluster 2 generally evidenced more positive CBRS. Patients in the *‘*low symptom-focussing and avoidance’ group did not engage as much as patients in the ‘high avoidance’ cluster, in unhelpful cognitive responses and exhibited less fear avoidance beliefs, beliefs that symptoms were damaging, and focussing on symptoms. Patients in Cluster 2 scored lower in behavioural responses to symptoms (resting and all-or-nothing). Additionally, patients in the ‘low symptom-focussing and avoidance’ group scored much lower than patients in the ‘high avoidance’ cluster on avoidance behaviours.

### Relationship between illness representation clusters and CBRS clusters with quality of life, depression and anxiety

Preliminary analysis was first conducted using independent samples t-tests to examine significant differences in QoL, depression and anxiety between clusters. When examining CBRS Clusters, patients in Cluster 1, ‘high avoidance’, had significantly lower QoL (*M* = 40.36, SD = 18.40) than patients in Cluster 2 ‘low symptom focussing and avoidance’ (*M* = 59.03, SD = 20.12); (*t*(184) = − 6.72, *p* < 0.001). Patients in Cluster 1 also had significantly higher depression (*M* = 7.20, SD = 5.71) compared with patients in Cluster 2 (*M* = 3.53, SD = 3.56); (*t*(189) = 3.68, *p* < 0.001). According to clinical-cut-offs [25], Cluster 1 patients on average indicated clinically mild symptoms of depression (scores between 5 and 9), compared to patients in Cluster 1 who, on average, had minimal symptoms of depression (scores between 0 and 4). Similarly patients in Cluster 1 had significantly higher anxiety (*M* = 5.70, SD = 5.90) compared with patients in Cluster 2 (*M* = 2.56, SD = 3.56); (*t*(189) = 3.35, *p* = 0.01). According to suggested clinical cut-offs [27] patients in Cluster 1 on average scored clinically moderate anxiety symptoms (scoring 5–9) compared with patients in Cluster 1 who on average had minimal anxiety symptoms (scoring 0–4).

Preliminary analysis was also conducted to examine significant differences between illness representation clusters with outcomes (QoL, anxiety and depression). Independent samples *t*-tests found that patients in illness perception Cluster 2, ‘negative illness and emotional representations’, had significantly lower QoL (*M* = 46.57, SD = 19.94) than in Cluster 1 ‘high coherence and treatment control’ (*M* = 61.52, SD = 21.38); (*t*(101) = − 4.55, *p* < 0.001). Patients in illness perception Cluster 2 also had significantly higher depression (*M* = 6.12, SD = 5.31) compared with patients in Cluster 1 (*M* = 2.85, SD = 2.97); (*t*(189) = 4.40, *p* < 0.001). According to clinical-cut-offs [25] Cluster 2 patients on average indicated mild symptoms of depression (scores between 5 and 9), compared to patients in Cluster 1 who, on average, had minimal symptoms of depression (scores between 0 and 4). Similarly patients in illness perception Cluster 2 had significantly higher anxiety (*M* = 4.70, SD = 5.27) compared with patients in Cluster 1 (*M* = 2.16, SD = 3.63); (*t*(189) = 4.55, *p* < 0.001). Patients in both Cluster 1 and 2 on average scored minimal anxiety symptoms (scoring 0–4) [27].

Hierarchical regression analyses were conducted relating to: (1) QoL, (2) depression, (3) anxiety. In each of the three regression models, the total overall variance explained (*R*^2^) is outlined, followed by examining the percentage variance explained by each block (block 1: clinical/demographic variables, block 2: illness representation cluster, and block 3: CBRS cluster) and an overview of the final model.

The variables in the first regression model (See Table 3) explained 29% of the variance overall in QoL (*F*(7, 185) = 10.88, *p* < 0.001, *R*^2^ = .29). In block 1, clinical and demographic variables significantly explained 11% of the variance in QoL, *F*(5,187) = 4.64, *p* = 0.001, *R*^2^ = 0.11. In block 2, illness representation cluster membership explained an additional 7.4% variance (*F*(6, 186) = 6.97, *p* < 0.001, *R*^2^ = .184; ∆*R*^2^ = 0.07, *p* < 0.001). In the third, final block, CBRS cluster significantly explained an additional 10.8% of the variance in QoL, over block 2 (*R*^2^ = 0.29; ∆*R*^2^ = 0.11, *p* < 0.001). Overall the whole model indicated that illness representations significantly contributed to the model: patients in the ‘high coherence and treatment control’ group (*M* = 61.52, SD = 21.38) reported a significantly higher QoL score, by 9.06 units, than patients in the ‘negative illness and emotional representations’ group (*M* = 46.57, SD = 19.94; *B* = 9.06, *t*(192) = 2.97, *p* = 0.03). Cognitive and behavioural responses to symptoms cluster also significantly contributed to the final model: Patients in the ‘low symptom-focussing, and avoidance’ group reported a significantly higher QoL score than patients in the ‘high avoidance’ cluster (*M* = 40.36, SD = 18.40) than patients in the ‘High avoidance and ‘low symptom-focussing, and avoidance’ cluster (*M* = 59.03, SD = 20.12) by 14.87 units (*B* = 14.87, *t*(192) = 5.41, *p* < 0.001).

**Table 3:** Hierarchical regression examining QoL

	*B* (S.E.)	Beta
*Block 1*		
Age	0.04 (0.18)	0.01
Gender (female)	− 15.64 (3.55)	− 0.31***
Previous cardioversions	1.17 (3.27)	0.03
Previous catheter ablations	6.80 (3.65)	0.13
Treatment procedure	1.42 (2.80)	0.04
*Block 2*		
Age	− 0.07 (0.17)	− 0.30
Gender (female)	− 13.38 (3.46)	− 3.87***
Previous cardioversions	1.56 (3.14)	0.04
Previous catheter ablations	5.02 (3.53)	0.10
Treatment procedure	2.08 (2.70)	0.06
Illness representation cluster	13.00 (3.17)	4.09***
*Block 3*		
Age	− 0.09 (0.16)	− 0.03
Gender (female)	− 11.77 (3.24)	− 0.24***
Previous cardioversions	0.53 (2.94)	0.01
Previous catheter ablations	3.83 (3.31)	0.08
Treatment procedure	2.68 (2.52)	0.07
Illness representation cluster	9.06 (3.05)	0.20**
CBRS cluster	14.87 (2.80)	0.34***

Block 1 *R*^2^ = 0.11, Block 2 *R*^2^ = 0.18, ∆*R*^2^ for block 2 = 0.07 (*p* < 0.001), ∆*R*^2^ for block 3 = 0.11 (*p* < 0.001)

*B* unstandardized regression coefficient, *S.E.* Standard error, *Beta* standardised regression coefficient

*p < 0.05, **p < 0.01, ***p < 0.001

The second regression (Table 4) explained 20.2% of the variance in symptoms of depression overall (*F*(7, 182) = 6.56, *p* < 0.001, *R*^2^ = 0.20). In block 1, clinical and demographic variables did not significantly contribute to the model (*F*(5, 184) = 1.68 *p* = 0.14). In block 2, illness representation cluster membership significantly explained 12.4% of the variance in depression (*F*(6,183) = 4.32, *p* < 0.001, *R*^2^ = .12; ∆*R*^2^ = 0.08, *p* < 0.001. In block 3, CBRS cluster significantly explained an additional 7.8% of the variance (*R*^2^ = .20; ∆*R*^2^ = 0.08, *p* < 0.001). Overall, the whole model indicated that illness representations significantly contributed to the model: patients in the ‘high coherence and treatment control’ group (*M* = 2.84, SD = 2.97) reported a significantly lower depression scores, by 2.22 units, than patients in the ‘negative illness and emotional representations’ group (*M* = 6.12, SD = 5.31, *B* = − 2.22, *t*(192) = − 3.07, *p* = 0.002). Cognitive and behavioural responses to symptoms clusters also significantly contributed to explaining the final model: Patients in the ‘low symptom-focussing and avoidance’ group reported a significantly lower depression scores (*M* = 3.53, SD = 3.56) than patients in the ‘high avoidance’ cluster (*M* = 7.20, SD = 5.71) by 2.81 units (*B* = − 2.81, *t*(189) = − 4.20, *p* < 0.001).

**Table 4:** Hierarchical regression examining depression

	*B* (S.E.)	Beta
*Block 1*		
Age	− 0.08 (0.04)	− 0.14
Gender (female)	1.72 (0.85)	0.15*
Previous cardioversions	− 0.78 (0.76)	− 0.08
Previous catheter ablations	− 0.11 (0.85)	− 0.01
Treatment procedure	− 0.31 (0.66)	− 0.04
*Block 2*		
Age	− 0.05 (0.40)	− 0.10
Gender (female)	1.23 (0.82)	0.11
Previous cardioversions	− 0.90 (0.73)	− 0.09
Previous catheter ablations	0.32 (0.82)	0.03
Treatment procedure	− 0.44 (0.63)	− 0.05
Illness representation cluster	− 3.00 (0.73)	− 0.29***
*Block 3*		
Age	− 0.05 (0.04)	− 0.09
Gender (female)	0.90 (0.79)	0.80
Previous cardioversions	− 0.74 (0.70)	− 0.08
Previous catheter ablations	0.58 (0.79)	0.05
Treatment procedure	− 0.51 (0.61)	− 0.06
Illness representation cluster	− 2.23 (0.73)	− 0.22**
CBRS cluster	− 2.81(0.67)	− 0.29***

Block 1 *R*^2^ = 0.04, Block 2 *R*^2^ = 0.12, ∆*R*^2^ for block 2 = 0.08 (*p* < 0.001), ∆*R*^2^ for block 3 = 0.08 (*p* < 0.001)

*B* unstandardized regression coefficient, *S.E.* standard error, *Beta* standardised regression coefficient

*p < 0.05, **p < 0.01, ***p < 0.001

The third regression model (Table 5) explained 14.7% of the variance in anxiety symptoms overall (*F*(7,182) = 4.47, *p* < 0.001, *R*^2^ = 0.15). In block 1, clinical and demographic variables did not significantly contribute to the model (*F*(5, 184) = 182, *p* = 0.11). In block 2, illness representation cluster significantly explained 8.9% of the variance (*F*(6, 183) = 2.98, *p* = 0.01, *R*^2^ = .09; ∆*R*^2^ = 0.04, *p* = 0.004). In block 3, CBRS cluster explained an additional 5.8% of the variance in anxiety (*R*^2^ = .15; ∆*R*^2^ = 0.06, *p* = 0.001). Overall, the whole model indicated that illness representations significantly contributed to the model: patients in the ‘high coherence and treatment control’ group (*M* = 2.16, SD = 3.63) had significantly lower anxiety scores, by 1.51 units, than patients in the ‘negative illness and emotional representations’ group (*M* = 4.70, SD = 5.27, *B* = − 1.51, *t*(189) = − 2.00, *p* < 0.05). Cognitive and behavioural responses to symptoms clusters also significantly contributed to explaining the final model for anxiety: Patients in the ‘low symptom-focussing and avoidance’ group reported a significantly lower anxiety scores (*M* = 2.56, SD = 3.56) than patients in the ‘high avoidance’ cluster scores (*M* = 5.70, SD = 5.90) by 2.45 units (*t*(189) = − 3.51, *p* = 0.01).

**Table 5:** Hierarchical regression examining anxiety

	*B* (S.E.)	Beta
*Block 1*		
Age	− 0.10 (0.04)	− 0.19**
Gender (female)	1.67 (0.85)	0.15
Previous cardioversions	− 0.77 (0.76)	− 0.08
Previous catheter ablations	0.19 (0.85)	0.02
Treatment procedure	0.46 (0.66)	0.05
*Block 2*		
Age	− 0.09 (0.04)	− 0.16*
Gender (female)	1.32 (0.84)	0.12
Previous cardioversions	− 0.86 (0.75)	− 0.09
Previous catheter ablations	0.51 (0.84)	0.04
Treatment procedure	0.37 (0.65)	0.04
Illness representation cluster	− 2.19 (0.75)	− 0.21*
*Block 3*		
Age	− 0.09 (0.40)	− 0.15*
Gender (female)	1.02 (0.82)	0.09
Previous cardioversions	− 0.72 (0.73)	− 0.07
Previous catheter ablations	0.73 (0.82)	0.06
Treatment procedure	0.31 (0.63)	0.04
Illness representation cluster	− 1.51 (0.76)	− 0.15*
CBRS cluster	− 2.45 (0.70)	− 0.25**

Block 1 *R*^2^ = 0.05, Block 2 *R*^2^ = 0.09, ∆*R*^2^ for block 2 = 0.04 (*p* = 0.004), ∆*R*^2^ for block 3 = 0.06 (*p* = 0.001)

*B* unstandardized regression coefficient, *S.E.* standard error, *Beta* standardised regression coefficient

*p < 0.05, **p < 0.01, ***p < 0.001

## Discussion

Two distinct clusters of illness representations and CBRS were found in AF patients. Cluster groups significantly differed in all CBRQ subscales. The majority of AF-IPQR subscales also significantly differed between cluster group, except timeline and procedural treatment control (although significant differences were found for pharmacological treatment control between clusters). For illness representations, the principle distinction between clusters related to high and lower beliefs about personal and pharmacological treatment control, AF triggers, and illness coherence. For CBRS, cluster membership was distinguished by high or lower fear avoidance and damaging beliefs, and engaging more or less in unhelpful behavioural responses such as all or nothing behaviour avoidance and excessive resting. Regressions indicated that both illness representation and CBRS cluster membership significantly contributed to explaining the variance in QoL, depression and anxiety, even after controlling for clinical and demographic factors (e.g. age, gender, treatments). The results support using clusters of illness representation clusters and CBRS clusters to identify patients at risk of adverse outcomes (discussed further below).

In relation to illness representations, around a third of patients were members of the ‘high coherence and treatment control’ group. These patients reported a good understanding of their AF (illness coherence) and beliefs that pharmacological treatment would be effective (treatment control beliefs). They attributed fewer symptoms to AF and believed that AF had fewer consequences and reported less negative emotional representations about AF. This cluster was associated with significantly better quality of life, lower anxiety and depression than patients in the ‘negative illness and emotional representations’ group.

More than two thirds of the sample held a more negative illness representation (‘negative illness and emotional representations). Patients in this cluster held beliefs that AF was cyclic, that they could personally control AF symptoms, and that overexertion (e.g. overwork and exercise), health behaviours (e.g. smoking and alcohol) and emotional factors (e.g. stress and emotional state) triggered AF symptoms. Patients in the ‘negative illness and emotional representations’ group also believed that AF had serious consequences on their lives and held negative emotional representations about illness.

While previous research has indicated that personal control is associated with more positive outcomes in people with chronic health conditions [15, 16, 19], in this study high personal control beliefs clustered with other more negative illness beliefs (Cluster 2). In the context of AF, higher personal control beliefs may be reflective of beliefs that AF is cyclic and that repeated behavioural and lifestyle modifications, reflected by high scores for this group on health-behaviour triggers, can prevent AF symptoms. This is supported by a recent qualitative study where patients who reported a perceived lack of understanding of AF, also tended to speak about increased monitoring of AF and control attempts, leading to increased emotional distress [37].

Previous cross-sectional studies which have looked at each illness perception dimension separately have also found that lower illness coherence is associated with psychological distress [38] and that beliefs that AF has greater consequences on everyday life, and attributing more symptoms to AF, are associated with poorer adjustment, psychological distress and poorer QoL [6, 38, 39]. The current study adds to these by showing a broader profile of beliefs which may be important. Ongoing attempts to control symptoms using methods which in fact do not control symptoms, such as avoiding exercise due to beliefs that overexertion may trigger AF, may lead to a vicious cycle of illness related distress and beliefs in the serious consequences of the condition.

Our results are also consistent with recent systematic review evidence which found significant relationships between illness representation schemata and health related outcomes in chronic illnesses [16]. Norton et al. [14] found two illness representation clusters in patients with rheumatoid arthritis, consisting of negative (high illness identity, consequences and chronic and cyclic timeline) and positive representations of illness, with negative cluster membership associated with high levels of pain, functional disability and distress. Similarly, Berry et al. [40] found three illness perception clusters in diabetes patients, associated with distress. Patients who believed diabetes had high consequences, was unpredictable and cyclic, and had negative emotions towards diabetes, had the highest level of distress, depression and greatest incidence of diabetes complications 12 months later. Our results differed slightly with McCorry et al. [15] who examined individuals with breast cancer. While illness representation schemata were broadly similar with the current study, McCorry et al. [15] found that greater personal control beliefs clustered together with more positive illness representation, such as fewer consequences, and that the illness would have a shorter timeline. This more positive cluster was significantly related to lower anxiety and distress. In the current study we found that greater personal control clustered with more negative illness representations, and was associated with poorer QoL and distress. As outlined above, personal control may be associated with beliefs that symptoms can be controlled. In chronic conditions such as AF where there is no cure, beliefs that symptoms can be managed through personal efforts may be detrimental leading to greater distress, compared to conditions where everyday symptoms can be more effectively managed, or in conditions with the potential for long-term cure.

In the current study, illness representation clusters only explained at most, an additional 12.4% of the variance in outcome (i.e. in relation to depression). A significant proportion of the variance in outcome was also explained by CBRS, in some cases explaining an additional 10.8% of the variance (i.e. for QoL). This provides support for the CSM framework, which outlines the importance of both behavioural coping responses to illness, as well as illness representations, which have thus far not been studied to the same extent.

Whilst there is no research outlining clusters of CBRS, several studies have examined individual CBRS in non-cardiac populations. In patients with end-stage kidney disease, all-or-nothing behaviours and avoidance behaviours explained a significant amount of the variance in fatigue [41]. Similarly, in patients with multiple sclerosis, all-or-nothing behaviours, avoidance and excessive rest predicted greater disability and fatigue [21]. Loades et al. [42] outlined that unhelpful CBRS, characterised by higher scores on all subscales of the CBRQ, were highly prevalent in patients with chronic fatigue syndrome (CFS), as in the current sample of AF patients, with approximately half AF patients in the ‘high avoidance’ cluster. In particular in CFS patients, damage beliefs, catastrophising and all-or-nothing behaviour predicted physical functioning. The authors suggested that patients who believe symptoms are damaging (damaging beliefs) or that doing more will exacerbate symptoms (fear avoidance) subsequently respond to symptoms with inactivity and avoidance (avoidance/rest). This inactivity may result in physical deconditioning, which exacerbate symptoms when activity is resumed [42]. As found in the ‘high avoidance’ cluster in the current study, patients engaging more in damaging and fear avoidance beliefs, also engage more in symptom focusing, avoidance behaviours, all-or-nothing behaviours and excessive rest, and have poorer QoL, and higher anxiety and depression. It may be that patients with a greater focus on symptoms respond more everyday variations in symptoms, some of which may not relate to AF. Responding to a wide range of symptoms could result in greater perceived impact of illness, impaired adjustment, poorer QoL, and greater distress. Conversely, engaging less in avoidance, all-or-nothing behaviours and excessive resting, and developing more consistent behavioural responses to symptoms could be associated with better outcomes. These suggestions are in line with the CBRS clusters; the ‘low symptom-focussing and avoidance’ group characterised by lower scores on symptom-damaging beliefs than patients in the ‘high avoidance’ cluster, and less reports of unhelpful behaviours, particularly excessive rest. The ‘low symptom-focussing and avoidance’ group patients and had significantly lower depression, anxiety and higher QoL than patients in the ‘high avoidance’ cluster.

Whilst previous research has also shown that negative beliefs about AF are associated with outcome, this is the first study to suggest the importance of day-to-day CBRS. It may be that patients’ day-to-day responses are more relevant in impacting outcomes, particularly in LTCs where there may be great variation and unpredictability in symptomatic-experience over time, such as in AF. To fully test the dynamic, self-regulatory CSM process, further longitudinal research is required.

One limitation of the study included the cross-sectional design, meaning that causal relationships cannot be drawn. There are also limitations of cluster analysis. For instance, the most important individual factors, contributing to explaining outcome, cannot be identified. Methods of clustering have also been criticised. For example, *K*-means analysis requires the number of clusters to be pre-set, and subsequent results are sensitive to these clusters [43]. Conversely, Ward’s method is more complex but requires a degree of subjectivity when interpreting clusters. To limit the weaknesses of both techniques, a two-step method was used, providing a more robust justification for the analysis, using hierarchical and non-hierarchical methods [13]. Another limitation of the study was that the sample consisted of a diverse range of patients taking different pharmacological and procedural treatments, which may have influenced reported beliefs about illness. Additionally, only less than a third of the variance in QoL, anxiety and depression were accounted for in the current models. Therefore, further research should examine other factors such as symptom severity and ongoing/current treatment which may account for additional variance. Furthermore, while individuals with more severe co-morbidities were not included in the study, individuals with common-comorbid conditions were eligible to participate. These common comorbid conditions, such as coronary artery disease and chronic obstructive pulmonary disease, which are associated with poorer QoL in AF [44], were not independently examined. However one key strength of the study was that we controlled for a range of important demographic and clinical variables in the regression.

Developing patient profiles using cluster analysis, and identifying whether clusters are related to adverse outcomes, enables clinicians to target patients in a time-efficient, relatively simple way (i.e. completing the IPQ-R and CBRQ during clinic visits). It is also likely that patients in different clusters will require different treatments. AF patients in the ‘negative illness and emotional representations’ group may benefit from interventions targeting education around AF, and how to manage symptoms and treatment, to improve emotions about AF.

Overall, this study found that AF patients’ illness beliefs and behavioural responses to symptoms, can be clustered to form two broad schemas. Further research should examine other factors, such as symptom severity or treatment plan, which may contribute to poorer adjustment, whether clusters found in the current study can predict QoL, depression and anxiety over time, and be used to identify patients at increased risk of poorer outcomes.

## Data Availability

The data that support the findings of this study are available on request from the corresponding author. The data are not publicly available due to privacy or ethical restrictions.

## References

[1] Patel D, Mc Conkey ND, Sohaney R, Mc Neil A, Jedrzejczyk A, Armaganijan L (2013). A systematic review of depression and anxiety in patients with atrial fibrillation: The mind-heart link. Cardiovascular Psychiatry and Neurology.

[2] Thrall G, Lip GY, Carroll D, Lane D (2007). Depression, anxiety, and quality of life in patients with atrial fibrillation. Chest.

[3] Dorian P, Paquette M, Newman D, Green M, Connolly SJ, Talajic M, Roy D (2002). Quality of life improves with treatment in the Canadian Trial of Atrial Fibrillation. American Heart Journal.

[4] Kirchhof, P., Bernussi, S., Kotecha, D., & Heidbuchel, H. (2017). ESC guidelines for the management of atrial fibrillation developed in collaboration with EACTS. *Revista española de cardiologia.-Madrid, 70*(1), 1–84. 10.1016/j.rec.2016.11.033 28038729

[5] Thrall, G., Lane, D., Carroll, D., & Lip, G. Y. (2006). Quality of life in patients with atrial fibrillation: A systematic review. *The American Journal of Medicine, 119*(5), 448. e441–e448. e419. 10.1016/j.amjmed.2005.10.057 16651058

[6] Taylor EC, O’Neill M, Hughes LD, Moss-Morris R (2018). An illness-specific version of the Revised Illness Perception Questionnaire in patients with atrial fibrillation (AF IPQ-R): Unpacking beliefs about treatment control, personal control and symptom triggers. Psychology & Health.

[7] Leventhal, H., & Brissette, I. (2003). The Common-Sense Model of self-regulation of health and illness. In L. D. Cameron, & H. Leventhal (Eds.), *The self-regulation of health and illness behaviour*. Routledge.

[8] Leventhal H, Meyer D, Nerenz D (1980). The common sense representation of illness danger. Contributions to Medical Psychology.

[9] French DP, Cooper A, Weinman J (2006). Illness perceptions predict attendance at cardiac rehabilitation following acute myocardial infarction: A systematic review with meta-analysis. Journal of Psychosomatic Research.

[10] Ross S, Walker A, MacLeod MJ (2004). Patient compliance in hypertension: Role of illness perceptions and treatment beliefs. Journal of Human Hypertension.

[11] Schoormans D, Mulder BJ, van Melle JP, Pieper PG, van Dijk AP, Sieswerda GT, Hulsbergen-Zwarts MS, Plokker THWM, Brunninkhuis LGH, Vliegen HW, Sprangers MAG (2014). Illness perceptions of adults with congenital heart disease and their predictive value for quality of life two years later. European Journal of Cardiovascular Nursing.

[12] Moss-Morris R, Weinman J, Petrie K, Horne R, Cameron L, Buick D (2002). The revised illness perception questionnaire (IPQ-R). Psychology and Health.

[13] Clatworthy J, Hankins M, Buick D, Weinman J, Horne R (2007). Cluster analysis in illness perception research: A Monte Carlo study to identify the most appropriate method. Psychology and Health.

[14] Norton S, Hughes LD, Chilcot J, Sacker A, van Os S, Young A, Done J (2014). Negative and positive illness representations of rheumatoid arthritis: A latent profile analysis. Journal of Behavioral Medicine.

[15] McCorry NK, Dempster M, Quinn J, Hogg A, Newell J, Moore M, Kelly S, Kirk KJ (2013). Illness perception clusters at diagnosis predict psychological distress among women with breast cancer at 6 months post diagnosis. Psycho-Oncology.

[16] Rivera E, Corte C, DeVon HA, Collins EG, Steffen A (2020). A systematic review of illness representation clusters in chronic conditions. Research in Nursing & Health.

[17] Biggs A, Brough P, Drummond S, Cooper CL, Quick JC (2017). Lazarus and Folkman’s psychological stress and coping theory. The handbook of stress and health: A guide to research and practice.

[18] Lazarus, R. S., & Folkman, S. (1984). *Stress, appraisal, and coping*. Springer publishing company.

[19] Hagger MS, Koch S, Chatzisarantis NL, Orbell S (2017). The common sense model of self-regulation: Meta-analysis and test of a process model. Psychological Bulletin.

[20] Hindricks G, Potpara T, Dagres N, Arbelo E, Bax JJ, Blomström-Lundqvist C (2021). 2020 ESC Guidelines for the diagnosis and management of atrial fibrillation developed in collaboration with the European Association for Cardio-Thoracic Surgery (EACTS) The Task Force for the diagnosis and management of atrial fibrillation of the European Society of Cardiology (ESC) Developed with the special contribution of the European Heart Rhythm Association (EHRA) of the ESC. European Heart Journal.

[21] Skerrett TN, Moss-Morris R (2006). Fatigue and social impairment in multiple sclerosis: The role of patients’ cognitive and behavioral responses to their symptoms. Journal of Psychosomatic Research.

[22] Ryan EG, Vitoratou S, Goldsmith KA, Chalder T (2018). Psychometric properties and factor structure of a long and shortened version of the cognitive and behavioural responses questionnaire. Psychosomatic Medicine.

[23] Spertus, J., Dorian, P., Bubien, R., Lewis, S., Godejohn, D., Reynolds, M. R., Lakkireddt, D. R., Wimmer, A. P., Bhandari, A., & Burk, C. (2011). Development and validation of the Atrial Fibrillation Effect on QualiTy-of-Life (AFEQT) Questionnaire in patients with atrial fibrillation. *Circulation: Arrhythmia and Electrophysiology, 4*(1), 15–25. 10.1161/CIRCEP.110.958033 21160035

[24] Dorian, P., Burk, C., Mullin, C. M., Bubien, R., Godejohn, D., Reynolds, M. R., Lakkireddy, D. R., Wimmer, A. P., Bhandari, A., & Spertus, J. (2013). Interpreting changes in quality of life in atrial fibrillation: how much change is meaningful? *American Heart Journal, 166*(2), 381–387. e388. 10.1016/j.ahj.2013.04.015 23895823

[25] Kroenke K, Strine TW, Spitzer RL, Williams JB, Berry JT, Mokdad AH (2009). The PHQ-8 as a measure of current depression in the general population. Journal of Affective Disorders.

[26] Kroenke K, Spitzer RL (2002). The PHQ-9: A new depression diagnostic and severity measure. Psychiatric Annals.

[27] Spitzer RL, Kroenke K, Williams JB, Löwe B (2006). A brief measure for assessing generalized anxiety disorder: The GAD-7. Archives of Internal Medicine.

[28] Cohen, J. (1988). *1988: Statistical power analysis for the behavioural sciences*. Erlbaum.

[29] Mehrotra DV, Liu F, Permutt T (2017). Missing data in clinical trials: Control-based mean imputation and sensitivity analysis. Pharmaceutical Statistics.

[30] Ward JH, Hook ME (1963). Application of an hierarchical grouping procedure to a problem of grouping profiles. Educational and Psychological Measurement.

[31] Everitt BS, Landau S, Leese M, Stahl D (2011). Cluster analysis.

[32] Hair, J. F. (2009). *Multivariate data analysis: A global perspective* (7th ed.). Prentice Hall.

[33] Bennasar-Veny M, Yañez AM, Pericas J, Ballester L, Fernandez-Dominguez JC, Tauler P (2020). Cluster analysis of health-related lifestyles in university students. International Journal of Environmental Research and Public Health.

[34] Kent P, Jensen RK, Kongsted A (2014). A comparison of three clustering methods for finding subgroups in MRI, SMS or clinical data: SPSS TwoStep Cluster analysis, Latent Gold and SNOB. BMC Medical Research Methodology.

[35] Gelbard R, Goldman O, Spiegler I (2007). Investigating diversity of clustering methods: An empirical comparison. Data & Knowledge Engineering.

[36] Benassi, M., Garofalo, S., Ambrosini, F., Sant’Angelo, R. P., Raggini, R., De Paoli, G. (2020). Using two-step cluster analysis and latent class cluster analysis to classify the cognitive heterogeneity of cross-diagnostic psychiatric inpatients. Frontiers in Psychology.

[37] Taylor EC, O'Neill M, Hughes LD, Carroll S, Moss-Morris R (2018). ‘It's like a frog leaping about in your chest’: Illness and treatment perceptions in persistent atrial fibrillation. British Journal of Health Psychology.

[38] McCabe PJ, Barnason SA (2012). Illness perceptions, coping strategies, and symptoms contribute to psychological distress in patients with recurrent symptomatic atrial fibrillation. Journal of Cardiovascular Nursing.

[39] Steed L, Newman S, Hardman S (1999). An examination of the self-regulation model in atrial fibrillation. British Journal of Health Psychology.

[40] Berry E, Davies M, Dempster M (2017). Illness perception clusters and relationship quality are associated with diabetes distress in adults with Type 2 diabetes. Psychology, Health & Medicine.

[41] Chilcot J, Moss-Morris R, Artom M, Harden L, Picariello F, Hughes H (2016). Psychosocial and clinical correlates of fatigue in haemodialysis patients: The importance of patients’ illness cognitions and behaviours. International Journal of Behavioral Medicine.

[42] Loades ME, Rimes K, Lievesley K, Ali S, Chalder T (2019). Cognitive and behavioural responses to symptoms in adolescents with chronic fatigue syndrome: A case–control study nested within a cohort. Clinical Child Psychology and Psychiatry.

[43] Xu D, Tian Y (2015). A comprehensive survey of clustering algorithms. Annals of Data Science.

[44] Randolph TC, Simon DN, Thomas L, Allen LA, Fonarow GC, Gersh BJ (2016). Patient factors associated with quality of life in atrial fibrillation. American Heart Journal.

